# Causal relationship between PCSK9 inhibitor and primary glomerular disease: a drug target Mendelian randomization study

**DOI:** 10.3389/fendo.2024.1335489

**Published:** 2024-03-06

**Authors:** Hangyu Duan, Yue Shi, Qi Zhang, Xiujie Shi, Yifan Zhang, Jing Liu, Yu Zhang

**Affiliations:** Xiyuan Hospital, China Academy of Chinese Medical Sciences, Beijing, China

**Keywords:** drug-target Mendelian randomization, pcsk9, HMGCR, IgAN, primary glomerular disease

## Abstract

**Background:**

Successive observational studies have highlighted low-density lipoprotein cholesterol (LDL-C) as a standalone risk factor for the progression of chronic kidney disease (CKD) to end-stage renal disease. Lowering LDL-C levels significantly reduces the incidence of atherosclerotic events in patients with progressive CKD. Recent research indicates that proprotein convertase subtilisin kexin 9 (PCSK9) inhibitors not only effectively lower LDL-C levels in CKD patients but also exhibit therapeutic potential for autoimmune diseases such as systemic lupus erythematosus, rheumatoid arthritis, and ulcerative colitis. However, the role of PCSK9 inhibitors (PCSK9i) in treating CKD beyond lowering LDL-C levels remains uncertain. Therefore, this study employs drug-targeted Mendelian randomization (MR) to investigate the causal impact of PCSK9i on primary glomerular diseases such as IgA nephropathy (IgAN), membranous nephropathy (MN), and nephrotic syndrome (NS).

**Methods:**

Single-nucleotide polymorphisms (SNPs) linked to LDL-C were sourced from the Global Lipids Genetics Consortium genome-wide association study (GWAS). Genes situated in proximity to 3-Hydroxy-3-methylglutaryl-coenzyme A reductase (HMGCR), and PCSK9 served as proxies for therapeutic inhibition of these targets. The causal link between PCSK9i and the risk of primary glomerular disorders was discovered using drug-target MR studies. The HMGCR inhibitor, a drug target of statins, was utilized for comparative analysis with PCSK9i. Primary outcomes included the risk assessment for IgAN, MN, and NS, using the risk of coronary heart disease as a positive control.

**Results:**

The inhibition of PCSK9, as proxied genetically, was found to significantly reduce the risk of IgAN [odds ratio, OR (95% confidence interval, CI) = 0.05 (−1.82 to 1.93), *p* = 2.10 × 10^−3^]. Conversely, this inhibition was associated with an increased risk of NS [OR (95% CI) = 1.78 (1.34–2.22), *p* = 0.01]. Similarly, HMGCR inhibitors (HMGCRi) demonstrated a potential reduction in the risk of IgAN [OR (95%CI) = 0.0032 (−3.58 to 3.59), *p* = 1.60 × 10^−3)^.

**Conclusions:**

PCSK9i markedly decreased the risk of IgAN, suggesting a potential mechanism beyond their primary effect on LDL-C. However, these inhibitors were also associated with an increased risk of NS. On the other hand, HMGCRi appears to serve as a protective factor against IgAN. Conversely, PCSK9i may pose a risk factor for NS, suggesting the necessity for cautious application and further research into their impacts on various glomerular diseases.

## Background

Glomerulonephritis, predominantly affecting young individuals, often leads to chronic kidney disease (CKD) and, ultimately, end-stage renal failure, contributing to significant morbidity and societal costs. The management of glomerulonephritis presents a formidable clinical challenge, necessitating prolonged immune regulation and immunosuppressive therapy. Such treatments, often involving long-term steroid use, are associated with severe side effects and limited symptomatic improvement. Consequently, unraveling the fundamental disease mechanisms and identifying new treatment targets for more effective prevention and treatment of primary glomerular diseases are of paramount importance. Studies indicate heightened susceptibility to dyslipidemia in patients with primary glomerular diseases, such as IgA nephropathy (IgAN) and membranous nephropathy (MN), with lipid-lowering showing promise in improving IgAN prognosis (1). Thus, dyslipidemia could have a vital part in the development and advancement of these conditions, warranting further exploration of the impact of various lipid-reducing medications on primary glomerular diseases.

Proprotein convertase subtilisin kexin 9 (PCSK9), a serine protease, is pivotal in regulating low-density lipoprotein cholesterol (LDL-C) processing and has emerged as a primary target for cholesterol-reducing therapies. While the heart-protective benefits of PCSK9 inhibitors (PCSK9i) are well established, their impact on primary glomerular diseases remains less clear. Clinical findings suggest that evolocumab, a fully human monoclonal antibody–targeting PCSK9, reduces cardiovascular outcomes in advanced CKD patients (2). Moreover, PCSK9i may influence genes related to cytokines and T-cell factors through synthetic sgRNA complementary to specific gene sequences, utilizing Cas9, an RNA-guided DNA nuclease (3). IgAN, an autoimmune disease characterized by circulating immune complex formation, currently lacks disease-specific treatment (4). Similarly, MN, marked by immune complex deposition in glomerular capillary walls, primarily relies on supportive care focused on mitigating non-specific renal insults. The role of immunosuppression, particularly systemic high-dose corticosteroid therapy, remains debatable and should be cautiously considered (5). Notably, increased PCSK9 expression correlates with CKD-related dyslipidemia, with emerging research suggesting a possible connection between PCSK9 and the immune function. PCSK9i are hypothesized to exert pleiotropic effects beyond lipid reduction (6). Compared to conventional lipid-reducing agents such as statins [3-Hydroxy-3-methylglutaryl-coenzyme A reductase inhibitors (HMGCRi)], PCSK9i may modulate immune responses by inhibiting dendritic cell-mediated T-cell activation (7). Furthermore, PCSK9i can ameliorate inflammation, oxidative stress, and autophagy, counteracting IL-6–induced inflammasome activation, autophagic cell accumulation, and mitochondrial reactive oxygen species (ROS) buildup (8). These findings imply PCSK9i involvement in autoimmune disease pathogenesis through non-lipid–lowering pathways. However, PCSK9i effects on autoimmune kidney diseases appear varied, necessitating further research.

This study utilizes drug-target MR analysis, employing genetic variations that mimic pharmacological target inhibition as instrumental variables. Through regression analysis, MR elucidates prolonged drug effects and strengthens cause and effect inference regarding drug genetic targets’ potential impact on autoimmune disorders (9, 10). We collected genome-wide association study (GWAS) statistics to examine the causal connection between genetically inferred PCSK9i and HMGCRi and primary glomerular diseases, including IgAN, MN, and NS, using a drug-targeted MR strategy.

## Methods

### Choosing of PCSK9 and HMGCR instrumental variables

The LDL-C data were sourced from GWAS summary statistics encompassing 72,866 East Asian individuals. Instrumental variables capable of targeting PCSK9 and HMGCR in reducing LDL-C levels were identified to mimic the effects of PCSK9i and HMGCRi (statins) (11). These instrumental variables comprised single-nucleotide polymorphisms (SNPs) situated within ±100 kb of the PCSK9 or HMGCR loci and associated with LDL-C levels. To mitigate the influence of strong linkage disequilibrium (LD) on the findings, an LD threshold was established (*r* ^2^ < 0.3). Subsequently, 32 significant SNPs linked to PCSK9 and 12 significant SNPs associated with HMGCR were preserved.

### Source of outcomes

The drug-target Mendelian randomization (MR) analysis utilized datasets for four diseases, with coronary heart disease (CHD) serving as a positive control. These datasets were exclusively derived from the East Asian population to maintain demographic consistency. The CHD dataset was procured from GWAS summary statistics, encompassing 60,801 cases and 123,504 controls (12). Additionally, summary datasets from GWAS for IgAN, MN, and nephrotic syndrome were also collated, serving as the primary outcomes for the study.

### Data analysis

PCSK9i and HMGCRi, integral in CHD treatment, allowed us to leverage the GWAS summary data of CHD as a positive control to affirm the efficacy of the instrumental variables. At the outset, we aligned the drug-targeting instrumental variables related to exposure with the datasets detailing outcomes. Subsequent analysis employed multiple methodologies, such as MR Egger, weighted median, inverse variance weighted (IVW), simple mode, weighted mode, and Mendelian Randomization Pleiotropy Residual Sum and Outlier (MR-PRESSO), with IVW emerging as the method most frequently applied (13).

Heterogeneity was assessed using the MR Egger and IVW approaches, using Cochrane’s Q statistic to assess the variability among the genetic instruments. A *p*-value exceeding 0.05 suggested the absence of significant heterogeneity. Furthermore, we employed the MR Egger regression method to examine the horizontal pleiotropy within the genetic instruments, where a *p*-value over 0.05 signifying an absence of horizontal pleiotropy (14).

**Figure 1:**
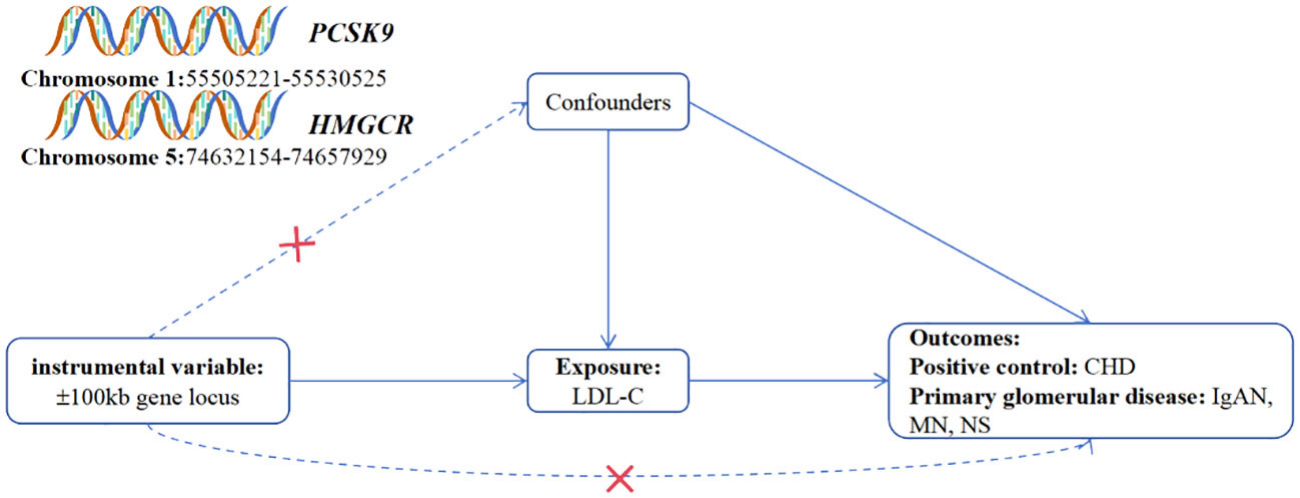
Overview of the research and design of drug target MR analysis. (1) The instrumental variables are not associated with the confounders (represented by dashed lines); (2) the instrumental variables are associated with the exposure factor (represented by solid lines); (3) the instrumental variables are not directly linked to the outcome (represented by dashed lines). LDL-C refers to low-density lipoprotein cholesterol; HMGCR denotes 3-hydroxy-3-methylglutaryl-coenzyme A reductase; PCSK9 stands for proprotein convertase subtilisin/kexin type 9; CHD represents coronary heart disease; IgAN refers to IgA Nephropathy; MN stands for membranous nephropathy; NS denotes nephrotic syndrome.

In compliance with the MR guideline stipulating that SNPs should not have a direct correlation with the outcome, the PhenoScanner website (http://www.phenoscanner.medschl.cam.ac.uk/) was employed to pinpoint traits directly associated with the SNPs serving as instrumental variables (**Figure 1**). SNPs associated with CHD, IgAN, MN, and NS were excluded. Post-exclusion, sensitivity analysis was reconducted using the MR-PRESSO test to remove any outliers. To ensure robustness against the influence of any specific SNP, the leave-one-out method was employed. This technique methodically excludes each SNP one by one, contrasting the results obtained from the IVW method when all variants are integrated. The analyses were carried out utilizing R version 4.3.1, supported by the MRPRESSO and TwoSampleMR packages (14, 15).

## Results

### Positive control analysis

**Figure 2:**
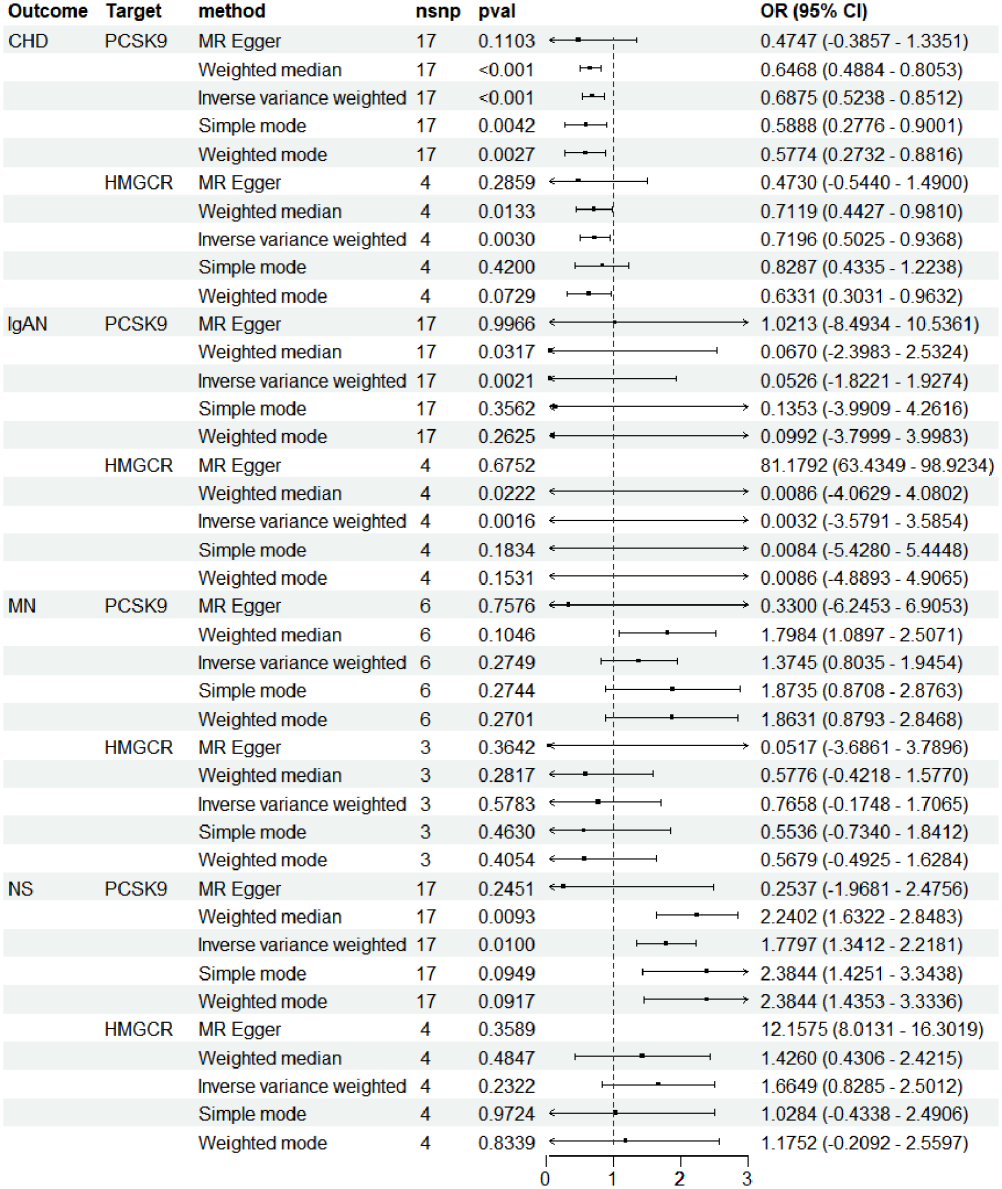
(Refer to the caption provided on the preceding page).

Consistent with expectations, outcomes from the IVW method revealed that PCSK9i markedly reduced the risk of CHD [OR (95%) = 0.69 (0.52 to 0.85), *p* < 1.0 × 10^−3^. This impact was akin to that observed with HMGCRi [OR (95%) = 0.72 (0.50 to 0.94), *p* = 3.0×10^−3^] (**Figure 2**). Additional analyses, employing methods such as MR Egger, simple mode, weighted mode, and MR-PRESSO, corroborated these findings, as detailed in Additional File 1. The robustness of these results was further validated by re-analyzing another GWAS dataset, which yielded similar outcomes.

### The causal link between genetically simulated inhibition of PCSK9 and HMGCR and conditions such as IgAN, MN, and NS

The genetic prediction of PCSK9i demonstrated a notable protective effect against IgAN, evidenced by the IVW approach [OR (95% CI) = 0.05 (−1.82 to 1.93), *p* = 2.1 × 10^−3^] and the weighted median method [OR (95% CI) = 0.07 (−2.40 to 2.53), *p* = 3.17 × 10^−2^). Inhibition of HMGCR also achieved statistical significance (IVW: *p* = 1.6 × 10^−3^; weighted median: *p* = 0.02) (**Figure 2**). Further outcomes from various MR analysis techniques are detailed in **Additional File 1**.

Conversely, genetic predictions indicated that HMGCRi had no significant impact on the risk of NS (IVW: *p* = 0.23; weighted median: *p* = 0.48). However, PCSK9i was positively associated with a heightened risk of NS, as shown by both the IVW method [OR (95% CI) = 1.78 (1.34–2.22), *p* = 0.01] and the weighted median method [OR(95% CI) = 2.24 (1.63–2.85), *p* = 9.3 × 10^−3^]. Moreover, PCSK9i and HMGCRi did not markedly influence the risk of MN (PCSK9i: IVW: *p* = 0.2; weighted median: *p* = 0.10; HMGCRi: IVW: *p* = 0.58; weighted median: *p* = 0.28).

### Sensitivity analysis

**Figure 3:**
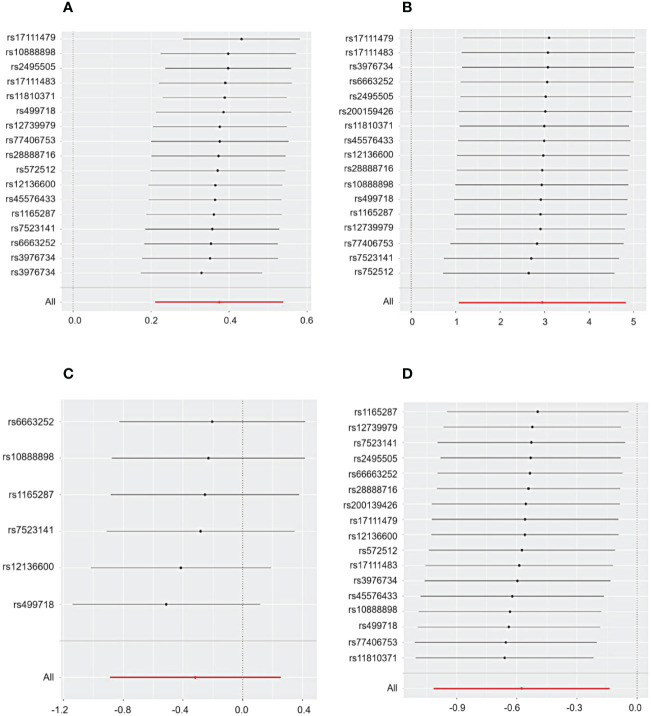
The sensitivity analysis for the effect of PCSK9 on CHD and IgAN, MN, and NS included a leave-one-out analysis focusing on **(A)** CHD, **(B)** IgAN, **(C)** MN, and **(D)** NS. The leave-one-out approach is employed to assess the undue influence of an individual SNP on MR analysis by determining if the aggregate effect of the remaining SNPs remains consistent with the primary effect after excluding one SNP.

**Figure 4:**
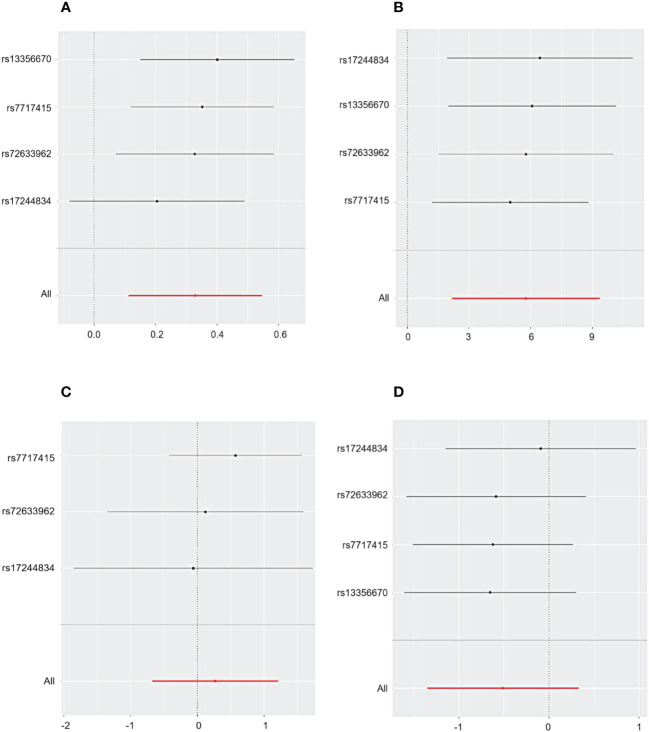
The sensitivity analysis for the influence of HMGCR on CHD, IgAN, MN, and NS includes a leave-one-out evaluation focusing on **(A)** CHD, **(B)** IgAN, **(C)** MN, and **(D)** NS.

Cochrane’s Q test and the MR Egger regression were employed to assess the degree of heterogeneity and horizontal pleiotropy, with detailed results provided in Additional File 1. The sensitivity analysis indicated no presence of heterogeneity or horizontal pleiotropy in relation to all outcomes, as denoted by *p*-values exceeding 0.05. Furthermore, the application of the leave-one-out method confirmed the robustness of our findings for CHD and primary glomerular diseases. This method demonstrated that omitting any single SNP did not lead to significant differences in the results, ensuring the reliability of our conclusions (**Figures 3**, **4**).

## Discussion

In this study, extensive genetic data were leveraged to uncover substantial evidence indicating that therapeutic suppression of the lipid-lowering drug target PCSK9 decreases the risk of IgAN, yet potentially heightens the risk of NS. The different roles of PCSK9i in different primary glomerular diseases may be due to differences in gene expression in these diseases.

PCSK9i has been well documented as beneficial for preventing and treating cardiovascular diseases (CVDs) due to its novel mechanism of lowering LDL-C levels (16, 17). However, the impact of PCSK9i goes beyond the mere reduction of LDL-C levels. Remarkably, PCSK9i demonstrates potential pleiotropic effects, including the improvement of tumor response to immune checkpoint therapy, reduction in platelet activation and thrombosis, and the attenuation of cell apoptosis (18–20). Recently, PCSK9’s role in inflammation and immunity, especially its contribution to the pathogenesis of autoimmune diseases, has attracted considerable interest (7, 21). Despite this, the direct causal relationship between PCSK9i and primary glomerular diseases has not been comprehensively explored. Our drug-target MR analysis revealed that PCSK9i markedly lowers the risk of IgAN but could increase the risk of NS. These results enhance our comprehension of the inflammatory impacts of PCSK9i, suggest possible side effects, and offer theoretical direction for choosing lipid-lowering approaches. Our results indicating a clear protective effect of PCSK9i on IgAN are particularly noteworthy. IgAN is distinguished by the presence of IgA-IgG immunodeposits within the glomeruli, exhibiting a mesangial distribution, which is thought to arise from circulating immune complexes. The underlying mechanisms of IgAN are hypothesized to be propelled by abnormal glycoforms of IgA1 (galactose-deficient IgA1, Gd-IgA1), which form circulating immune complexes upon recognition by IgG autoantibodies, leading to pathogenic IgA1-IgG immune complex formation. Activation of the complement system via alternative and/or lectin pathways is believed to significantly contribute to the pathogenic characteristics of these complexes, possibly intensifying local inflammatory reactions and causing further damage to the glomeruli (22). Plasma PCSK9 levels have been associated with various comorbidities, renal function indices, lipid parameters, and biomarkers of inflammation, oxidative stress, and endothelial damage (23). Studies have found contradictory relationships between PCSK9 levels and kidney disease. For instance, Konarzewski M et al. observed elevated circulating PCSK9 concentrations in patients with CKD without corresponding hypercholesterolemia (24). In contrast, Bermudez-Lopez M et al. reported an inverse relationship between PCSK9 levels and CKD stages, with a positive correlation between the PCSK9/LDL-C ratio and CKD stages (25). Vlad CE et al. noted significantly increased PCSK9 and hsCRP levels in patients newly diagnosed with kidney disease, linking elevated PCSK9 and hsCRP levels to earlier renal and cardiovascular events (26). Conversely, Shen H et al. reported significantly higher plasma PCSK9 levels in patients with primary nephrotic syndrome compared to healthy controls, a finding that contradicts our study (27). These conflicting results underscore the need for more extensive clinical and basic research on the effects of PCSK9i across different types of kidney diseases. There is a lack of clinical studies of PCSK9i in MN. Different primary glomerular diseases have different pathologic phenotypes and large differences in clinical prognosis, which may explain the different effects of PSCK9i on these diseases. Dyslipidemia is a risk factor for primary glomerular diseases, so lipid-lowering regimens for different types of primary glomerular diseases need to be further explored.

It must be acknowledged that this study comes with several significant limitations that warrant attention. First, MR analysis serves solely as a method to evaluate the causal link between exposure and outcome, and its scope is inherently limited. Second, the identified link between PCSK9i and the risk of primary glomerular diseases requires verification through additional research. Moreover, our MR analysis was confined to East Asian individuals, a limitation imposed by the lack of extensive GWAS data resources. Considering the genetic diversity across various ethnic populations, the effectiveness and potential side effects of PCSK9i might vary considerably. Consequently, it is imperative for future research to undertake subgroup analyses across varied populations, aiming to derive a more universally applicable and comprehensive conclusion.

## Conclusions

Through meticulous drug-target MR analysis, our study elucidates that the genetic forecast for PCSK9i notably decreases the risk of IgAN. However, this same genetic intervention appears to elevate the risk of NS. These findings underscore the nuanced and potentially dualistic nature of PCSK9 inhibitors’ effects on primary glomerular diseases, paving the way for more targeted and personalized approaches in managing these conditions.

## Data availability statement

The raw data supporting the conclusions of this article will be made available by the authors, without undue reservation. The authors affirm that all data underpinning the conclusions of this research are contained within the article and its additional files.

## Ethics statement

Ethical clearance for the use of GWAS summary data, which were procured from the online public repository (https://gwas.mrcieu.ac.uk/), was granted by relevant local ethics boards. Written consent was obtained from all participants involved in the study.

## Author contributions

HD: Investigation, Writing – original draft, Writing – review & editing. YS: Formal analysis, Writing – review & editing. QZ: Formal analysis, Writing – review & editing. XS: Data curation, Writing – review & editing. YiZ: Formal Analysis, Writing – review & editing. JL: Methodology, Writing – review & editing. YuZ: Supervision, Writing – review & editing.
